# The American Climate

**Published:** 1878-06

**Authors:** 


### THE AMERICAN CLIMATE.

" *To the Editor*

" Sir :—Having read an article in ' The Tribune' on the American climate, in which it is stated that an English Review and M. Deser have pronounced our climate unfavorable to physical vigor, I must beg permission to say a word on the subject.

" All portions of the earth have their own climate, made from a combination of causes, of which the proximity of water is one of the chief. In Western Europe the climate is what we see from its vicinity to the ocean on the frequency of westerly winds, in carrying the moisture landward ; for it is a fact well known to seamen that westerly winds prevail on the ocean, especially on the Pacific, from its greater extent probably, all but constantly above the region of the trade winds say from 25 to 30 deg., as easterly winds do within those paral lels. These westerly winds carry evaporation from our Atlantic coast, instead of bringing it to us, as in Europe. The fact that different countries fall on the same isothermal lines has little to do with their humidity. Ireland and England lie over against Labrador ; France corresponds with Canada and New England ; Spain and Morocco with our Middle and Southern States. Just go over the Pacific, and you will find at Vancouver's Island, and the country about Nootka Sound the same climate as in England and Holland, except what may be occasioned by the country back being high land, instead of low, like Holland. And is not the climate of Californi much like that of Spain ? It is, or should by this time be known, that these prevalent west winds north of 30 deg., give mildness of climate to the west sides of both continents, and that in the Eastern part of Europe the air is dry, and the summer and winter temperature more variable. Why, even here in Michigan, the open waters of Lake Michigan, in extreme winter weather, when westerly winds prevail, as they usually do, give us 10 deg. over Wisconsin and Northern Illinois.

" I was raised in New Hampshire, but have resided in Oregon, and have observed that the moisture naturally depends on the proximity of water, the course of the winds and the extent and elevation of the land, varying according as the thousands of locations vary as to surrounding objects, In Tartary and other parts of Central Asia, you find the climate of Utah, Nebraska and New Mexico, while the United States are not unlike China. All these circumstances modify the human and other animals the world over. The extremes of our winters and summers I suppose have more to do in forming the American character, than the dryness of the air; for in New England, with the sterile soil and severe winters, activity is necessary to existence, as it were, and the activity and industry thus induced have become habitual and constitutional. It may be with our extreme activity we may not last so long or be so fat as the more phlegmatic Europeans, though we should be compared with the French and Spaniards as to location, and not with the inhabitants of the British Islands and Northern Germany.

"Your obedient servant,

"Grand Rapids, Mich., Jan. 20, "J. B."

---

### PITCHING INTO NICODEMUS.

A CELEBRATED character of the State of New York, holding a high post in the law, was lately taken ill and confined to his bed for several days. His wife proposed to read for him to which he readily assented.

"My dear, what shall I read?"

"Oh, I don't care much what, anything you please."

"But have you no choice?"

"None in the world, love; please yourself."

"Shall I read a chapter or two out of the Scriptures?"

"Oh, yes, that'll do very well."

"But what part of the Scripture shall I read?"

"Any part you like, love."

"But, you must have some choice, some little preference; we all have that."

"No, I have none in the world; read any part you like best."

"But I would rather please you, dear, and you surely have a preference."

"Well, well, dear; if you will please me, then, *pitch into Nicodemus.*"

### SUGAR AND TEETH.

In a previous number it was stated that pure sugar and candies, having no residue, could not, by lodgement about the teeth, injure them; and that if used in moderation, neither sugar nor candies were prejudicial to the teeth or health of young children or grown persons; that there was more or less sugar in all vegetable food; but as concentrations were liable to abuse, we advised that they should be taken at regular meals.

The *Medical Journal* of Charleston, S. C., states the conclusions of M. Larez:

1st. Refined Sugar injures teeth, either by immediate con tact, or by gas developed in the stomach.

2d. That a tooth soaked in sugar water, becomes jelly like, from the sugar combining with the lime of the tooth.

